# Distribution of Non-Structural Carbohydrates and Root Structure of *Plantago lanceolata* L. under Different Defoliation Frequencies and Intensities

**DOI:** 10.3390/plants13192773

**Published:** 2024-10-03

**Authors:** Verónica M. Merino, René I. Aguilar, M. Jordana Rivero, Iván P. Ordóñez, Luis F. Piña, María Dolores López-Belchí, Mauricio I. Schoebitz, Felipe A. Noriega, Claudia I. Pérez, Andrew S. Cooke, Lubia M. Guedes

**Affiliations:** 1 Departamento de Producción Animal, Facultad de Agronomía, Universidad de Concepción, Casilla 160-C, Concepción 4030000, Chile; reaguilar.san@gmail.com; 2 Net Zero and Resilient Farming, Rothamsted Research, North Wyke, Okehampton EX20 2SB, Devon, UK; jordana.rivero-viera@rothamsted.ac.uk; 3 Instituto de Investigaciones Agropecuarias, INIA Kampenaike, Avenida España 01720, Punta Arenas 6200000, Chile; ivan.ordonez@inia.cl; 4 Departamento de Producción Animal, Facultad de Ciencias Agronómicas, Universidad de Chile, Santiago 8820808, Chile; luispina@uchile.cl; 5 Departamento de Producción Vegetal, Facultad de Agronomía, Universidad de Concepción, Av. Vicente Méndez 595, Chillán 3780000, Chile; mlopezb@udec.cl (M.D.L.-B.); fnoriega@udec.cl (F.A.N.); 6 Departamento de Suelos y Recursos Naturales, Facultad de Agronomía, Universidad de Concepción, Barrio Universitario s/n, Concepción 4030000, Chile; mschoebitz@udec.cl; 7 Departamento de Botánica, Facultad de Ciencias Naturales y Oceanográficas, Universidad de Concepción, Casilla 160-C, Concepción 4030000, Chile; claudiaperez@udec.cl; 8 Department of Life Sciences, School of Natural Sciences, College of Health and Science, University of Lincoln, Lincoln LN6 7DL, Lincolnshire, UK; ancooke@lincoln.ac.uk; 9 Laboratorio de Semioquímica Aplicada, Facultad de Ciencias Forestales, Universidad de Concepción, Casilla 160-C, Concepción 4030000, Chile

**Keywords:** plantain, defoliation management, extended leaf length, water-soluble carbohydrates, morphological composition, dry matter allocation, root structure

## Abstract

*Plantago lanceolata* L. (plantain) increases herbage dry matter (DM) production and quality during warm and dry conditions due to its deep roots and drought tolerance and reduces nitrogen losses in grazing systems compared to traditional pastures. However, plantain density usually declines after the third growing season, mainly due to defoliation management. The effects of defoliation frequency and intensity on water-soluble carbohydrate (WSC) reserves and below-ground plant responses need further research to optimize grazing strategies for improved productivity and sustainability of grazing systems. Our study investigated the effects of defoliation frequencies (15, 25, and 35 cm of extended leaf length, ELL) and intensities (5 and 8 cm of residual heights) on morphological traits and WSC concentrations in plantain biomass under controlled environmental conditions. Defoliation frequency significantly influenced morphological and chemical characteristics and biomass distribution more than residual height. Less frequent defoliations promoted above-ground herbage DM production, reproductive stems, and root biomass. Root architecture showed adaptations in response to defoliation frequency, optimizing resource acquisition efficiency. Frequent defoliation reduced high molecular weight WSC concentrations in leaves, affecting regrowth capacity and DM mass. A defoliation frequency of 25 cm ELL (~15 days) balances herbage production and root development, promoting long-term pasture sustainability.

## 1. Introduction

Perennial ryegrass (*Lolium perenne* L.) and white clover (*Trifolium repens* L.) are the main sown forage species in temperate regions. However, the increasing frequency and duration of soil moisture deficits from summer to autumn have led to herbage shortages in sown pastures, reducing animal production. This has led to the exploration of alternative forage species [1,2,3]. Among these, plantain (*Plantago lanceolata* L.; Plantaginaceae) has emerged as a promising option for grazing animals during drought periods [4,5] due to its greater ability to access soil water compared to perennial ryegrass [6].

Plantain is a perennial herb characterized by its deeper rooting system and greater drought tolerance than ryegrass and white clover species, which enhances herbage nutritive value and animal performance during dry months [7]. Moreover, recent research has highlighted the role of plantain in reducing N footprints from pasture-based production systems by lowering urinary N excretion [8] and soil nitrification [9]. Additionally, plantain has shown potential in reducing enteric methane emissions [10]. Despite these benefits, plantain’s persistence in permanent pastures declines after the second year due to factors such as plant competition, climatic stress, and defoliation management [10]. Therefore, understanding plantain’s response to these stresses is particularly important in the context of climate change, as this knowledge could help optimize grazing strategies, enhance pasture resilience, and support more sustainable livestock production systems.

Defoliation management, including defoliation frequency (time between successive defoliations) and defoliation intensity (amount of herbage mass removed or residual height) [11], influences the capacity to restore photosynthetic activity and replenish WSC reserves [4] and root growth [1]. Both traits, soil exploration [1,6] and WSC accumulation [12], are strongly correlated to plant growth and survival during soil moisture deficits. Forage species such as perennial ryegrass [13], praire grass (*Bromus willdenowii* Kunth [14,15], pasture brome (*B. valdivianus* Phil) [16,17], cocksfoot grass (*Dactylis glomerata* L.) [15,18,19], and chicory (*Cichorium intybus* L.) [20] exhibit similar responses when they are defoliated at high frequency or intensity, showing a reduction in both growth and WSC reserves. However, the plant development stage in which the WSC accumulation and the root and shoot growth are maximized differs among forage species [17,18]. Therefore, to achieve sustainable pasture utilization, it is essential to establish the appropriate defoliation criteria for each species of interest. In particular, for plantain, the impact of defoliation severity on the distribution of WSC reserves and below-ground responses remains unclear. Water soluble carbohydrates provide energy for winter survival and initiate leaf regrowth until sustained carbohydrate production by photosynthesis [19,20]. After defoliation, WSC reserves in roots and shoot tissues decrease to support shoot growth [4,14,21]. The developmental stage of plantain plants at the time of defoliation significantly influences the response to defoliation frequency [2,3]. 

Low defoliation frequencies result in leaf senescence and the development of reproductive stems, thereby reducing the leaf-to-stem ratio and herbage quality for ruminant nutrition [4,14,21]. Therefore, increasing defoliation frequency in plantain plants is advisable to improve pasture quality, enhancing herbage intake and animal yield [3]. However, frequent defoliation may hinder leaf elongation rate and root growth if there is not enough time to recover WSC reserves between cuts in successive rotations [4,22]. This could potentially lead to reduced plant survival and DM production, ultimately diminishing the persistence of sown species in permanent pastures over the long term [20].

Defoliation intensity has a lesser effect than defoliation frequency on herbage DM production, nutritional features, and the persistence of plantain pastures [4,23], as most WSC reserves are stored in the roots rather than the stubble [4,21]. The allocation of photoassimilates between above- and below-ground biomass, influenced by defoliation management, can lead to changes in plant architecture and root development and structure. For example, defoliation can result in variations in root length [1] and root DM accumulation [17], depending on the severity of defoliation. Plants grazed more frequently allocate greater resources to above-ground biomass, resulting in smaller taproots that increase susceptibility to death after defoliation [20]. Fine roots are crucial for resource extraction from the soil [24] and play a significant role in maintaining plant water status [25]. Taproots connect the fine roots to other plant organs and serve as storage for WSCs [26]. Fine roots have a greater demand for photoassimilates than taproots [27]. Therefore, it is reasonable to assume that under photosynthetic restriction, determined by defoliation frequency and intensity, fine roots would be the first to be affected, impacting root growth and development and, consequently, water and nutrient uptake capacity. 

The effect of grazing severity on the distribution of WSC reserves and below-ground responses in plantain pastures remains incompletely understood. Severe defoliation can lead to structural changes, including reductions in root diameter, radial root area, stele diameter, and vascular vessel numbers. These alterations could compromise root system development, limit nutrient uptake and exploration, and ultimately hinder new shoot growth [28]. However, there is a limited understanding of how defoliation management influences the length and width of vessel elements, which are crucial for water and nutrient transport from soil to aerial organs. A more comprehensive characterization of morphometric traits and the quantification and distribution of WSC reserves could provide valuable insights into the adaptive mechanisms of plantain under different defoliation regimes. This knowledge is essential for developing optimal grazing management strategies to enhance productivity and long-term persistence of plantain pastures [4,29]. Based on these gaps in knowledge, we propose the following research questions: (i) How do defoliation frequency and intensity affect DM distribution? (ii) How do defoliation frequency and intensity affect plantain root architecture? and (iii) What is the impact of defoliation frequency and intensity on WSC reserves in plantain? Our hypothesis is that under less severe defoliation regimes (i.e., with lower frequency and intensity of defoliation), the aerial and root growth, the WSC concentration, and the fine roots system of plantain will be enhanced. To test the hypothesis, the aim was to evaluate the effects of different defoliation frequencies (15, 25, and 35 cm of extended leaf length, ELL) and intensities (5 and 8 cm of residual heights) on the morphological features and carbohydrate concentration in both above- and below-ground DM mass of plantain plants. 

## 2. Results

### 2.1. Morphological Components and Above- and Below-Ground Biomass

**Figure 1:**
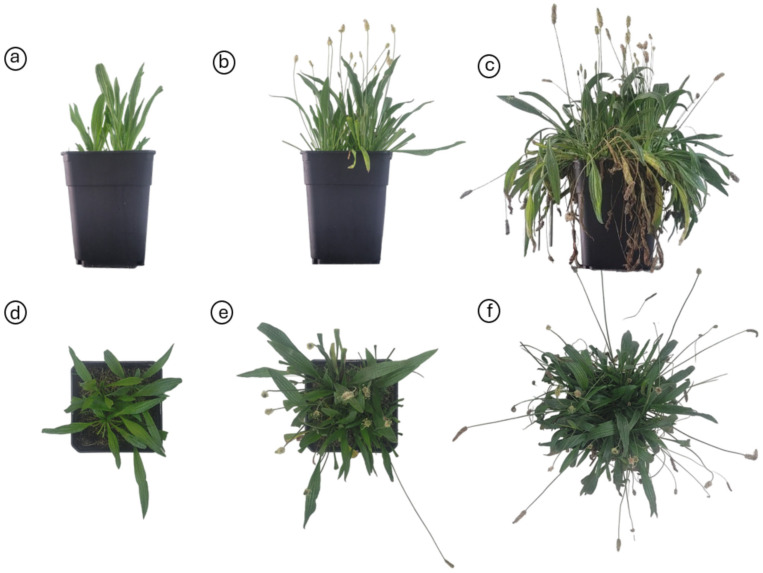
Pots in inside view (**a**–**c**) and top view (**d**–**f**) with *Plantago lanceolata* plants cut at different defoliation frequencies. (**a**–**d**) Defoliation at 15 cm of extended leaf length. (**b**–**e**) Defoliation at 25 cm of extended leaf length. (**c**–**f**) Defoliation at 35 cm of extended leaf length.

**Figure 2:**
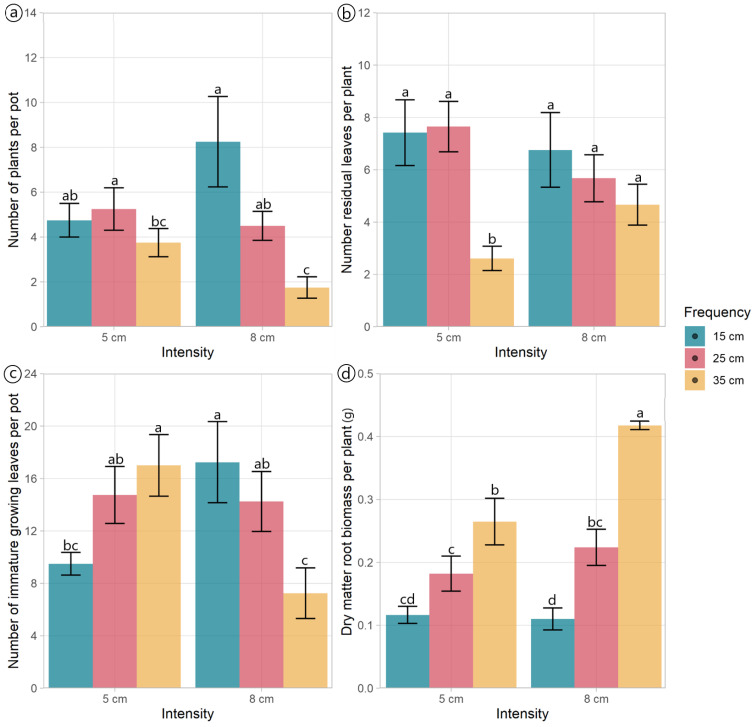
Morphological traits of *Plantago lanceolata* plants under three defoliation frequencies (15, 25, and 35 cm of extended leaf length) and two defoliation intensities (5 and 8 cm). Different letters indicate a significant difference between treatments for *p* < 0.05. Vertical bars represent Fisher’s least significant difference.

The morphological components and DM allocation plantain were mainly affected by defoliation frequency (Table 1, Figure 1). The number of plants per pot was affected by the interaction effect between defoliation frequency and intensity (*p* = 0.028) (Table 1). Plants defoliated at frequencies of 15 and 25 cm had similar numbers of plants per pot, independent of the defoliation intensity used, while the number of plants per pot cut to 8 cm decreased as the plants were defoliated less frequently (at 35 cm ELL) (Figure 2a). The defoliation frequency had an opposing effect on the total number of leaves and stems per pot (Figure 1). The number of leaves decreased (*p* = 0.01) when plants were defoliated less frequently (at 35 cm of ELL), while the number of stems increased (*p* = 0.003) (Figure 1).

The number of residual leaves per plant was affected by the interaction effect between defoliation frequency and intensity (*p* = 0.044). Plants defoliated to a residual height of 5 cm, and a frequency of 35 cm of ELL had the lowest number of residual leaves compared with all other treatments (Figure 2b). The number of residual leaves per pot was notably affected by the defoliation frequency (*p* < 0.001), with the plants defoliated at 35 cm of ELL containing less than a fourth of the number of leaves per pot compared to those subjected to the other two frequencies (Table 1). Both the number of fully expanded green leaves and growing leaves per plant varied with the defoliation frequency (*p* < 0.001). The highest values were observed at 35 cm of ELL, while the lowest values were detected at 15 cm of ELL (Table 1). The defoliation management did not affect the number of fully expanded leaves per pot (*p* > 0.05), averaging 15.1 leaves per pot. However, the number of growing leaves per pot exhibited an interaction effect (*p* = 0.001). While plants defoliated at 25 cm of ELL were unaffected by the defoliation intensity, plants defoliated at 15 cm and 35 cm of ELL had opposite responses to defoliation intensity (Figure 2c).

The DM mass of leaves per pot was lower (*p* = 0.021) under the defoliation frequency of 15 cm compared to 25 cm of ELL. However, there were no differences in the DM mass between plants defoliated at 35 cm of ELL and those treated with the other two defoliation frequencies (Table 1). Similarly, the DM mass of stems per pot was the lowest (*p* = 0.003) when plants were defoliated more frequently (at 15 cm of ELL), whilst the less frequent defoliations (25 cm and 35 cm of ELL) had higher values (Table 1). The dead DM per pot also increased (*p* < 0.001) when the defoliation frequency decreased (from 15 cm to 35 cm of ELL) (Table 1). In contrast, the defoliation frequency did not affect the root’s DM mass per pot (*p* = 0.084), averaging 0.81 g per pot. The above-ground biomass per plant increased (*p* = 0.002) when the defoliation frequency decreased (Table 1). The mean DM mass of roots per plant showed an interaction effect (*p* = 0.012); plants defoliated at 15 cm of ELL exhibited a reduction in root DM mass due to defoliation intensity (Figure 2d). The ratio between above-ground and root biomass was the only variable affected by the defoliation intensity (*p* = 0.025), with plants defoliated at 5 cm residual, showing a 32% increase in this ratio (Table 1). 

### 2.2. Root Morphology and Architectural Traits

**Figure 3:**
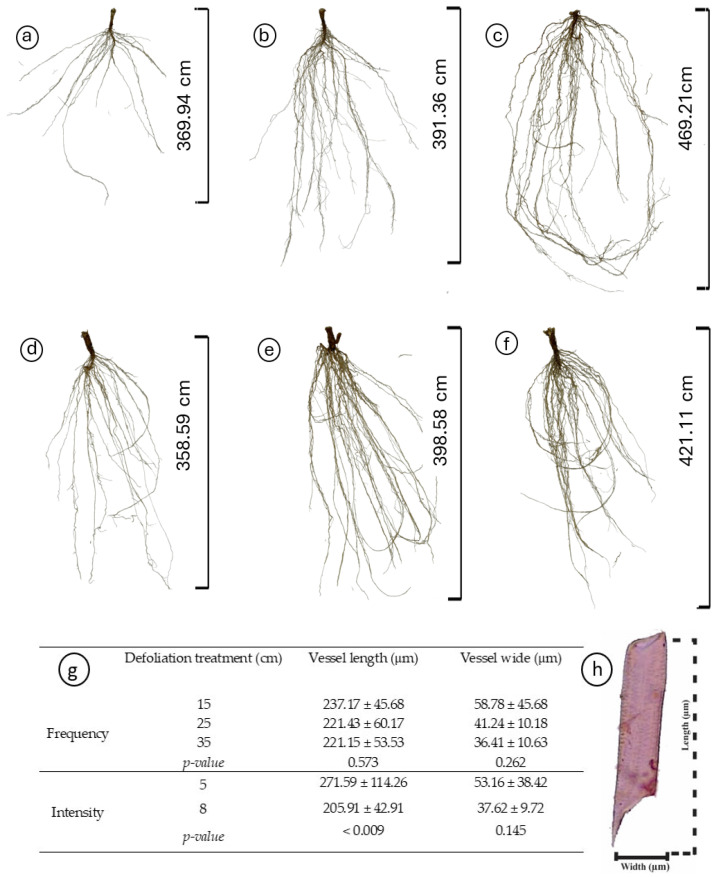
Impact of defoliation frequencies (15, 25, and 35 cm of extended leaf length) and intensities (5 and 8 cm) on the root morphology and vessel element dimensions of *Plantago lanceolata*. (**a**–**f**) Morphological variations in roots under different defoliation frequencies (15 cm in (**a**,**d**); 25 cm in (**b**,**e**); and 35 cm in (**c**,**f**)) and intensities (5 cm in (**a**–**c**), 8 cm in (**d**–**f**)); (**g**) Comparison of vessel element length and width across treatments, depicted with mean values and standard deviations; (**h**) Dimensions (width and length) of the root vessel elements. Significantly different treatments are denoted by different letters.

**Figure 4:**
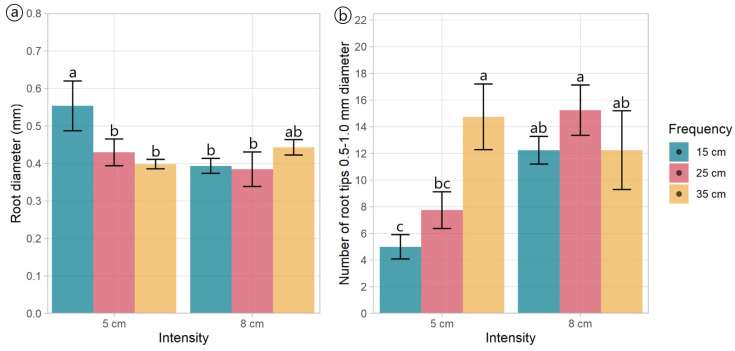
Root morphology and architectural traits of plants of *Plantago lanceolata* submitted to three defoliation frequencies (15, 25, and 35 cm of extended leaf length) and two defoliation intensities (5 and 8 cm): (**a**) Average diameter of roots and (**b**) Number of tips from 0.5 and 1.0 mm in diameter. Different letters indicate a significant difference between treatments for *p* < 0.05. Vertical bars represent Fisher’s least significant difference.

Regardless of the treatments, plantain roots exhibited a profuse root system, varying in architectural traits depending on the specific treatment conditions (Table 2, Figure 3). Root length varied with defoliation frequency (*p* = 0.014), with plants defoliated more frequently (at 15 cm of ELL) presenting shorter roots (Figure 3a,d) than those defoliated less frequently (25 and 35 cm of ELL) (Table 2, Figure 3b,c,e,f). Similarly, defoliation frequency had a negative effect (*p* = 0.047) on root surface area, with plants defoliated more frequently (at 15 cm of ELL) presenting smaller root areas (Table 2). In comparison, plants defoliated less frequently (at 35 cm of ELL) exhibited larger root areas, and the intermediate defoliation frequency (at 25 cm of ELL) presented the intermediate root area (Table 2, Figure 3). The average diameter was only affected by an interaction effect, where plants defoliated less frequently (at 15 cm of ELL) and at the lowest cut height (at 5 cm) had the thickets roots (Figure 4a), but similar to the other contrasting combinations (i.e., 35 cm of ELL frequency and 8 cm intensity). In contrast, all the different combinations had thinner roots (Figure 4a). However, root volume was not affected (*p* > 0.05) by any of the defoliations management, averaging 0.642 cm^3^ (Table 2). The number of root crossings, forks, and tips were not affected by any of the treatments (*p* > 0.05), averaging 322, 2423, and 1265, respectively (Table 2).

Most of the root tips (98.9%) belonged to roots thinner than 0.5 mm, while 0.911% belonged to roots with a diameter between 0.5 and 1.0 mm (Table 2, Figure S1), and this distribution was not affected by the defoliation management. The number of root tips with a diameter between 0.5 and 1.0 mm had an interaction effect (*p* = 0.02, Table 2) as follows: plants defoliated at 8 cm did not show an effect of the defoliation frequency, whereas plants defoliated at 5 cm did, where more frequent defoliation produced fewer root tips between 0.5 to 1.0 mm thickness (Figure 4b). Similar to the total root length and surface area, the more frequent defoliation produced shorter and smaller (less surface area) roots (*p* < 0.05) within the diameter classes 0–0.5 mm and 0.5–1.0 mm (Table 2, Figures S2 and S3). Unlike the total volume, the defoliation frequency affected the volume of the thinnest roots (*p* = 0.010). It tended (*p* = 0.063) to affect the volume of the roots within the diameter classes 0–0.5 mm and 0.5–1.0 mm, respectively (Table 2). This aligns with the larger volume of the roots with a diameter greater than 4.5 mm (Figure S4).

The dimensions of the vessel elements were only significantly affected by the defoliation intensity (*p* < 0.01) (Figure 3f). When the plants were cut at 8 cm, there was a significant decrease in the length of the vessel elements (Figure 3g).

### 2.3. Carbohydrates Concentration in Leaves and Roots

High-MWCs exhibited higher concentrations than low-MWCs in plantain leaves and roots (Table 3). The concentration of high-MWCs in the leaves was the only carbohydrate group affected by the defoliation management, specifically by the defoliation frequency (*p* = 0.044). Plants defoliated less frequently (at 35 cm of ELL) had a higher concentration compared to those defoliated more frequently (at 15 and 25 cm of ELL) (Table 3). The low-MWCs of leaves averaged 1.28 mg of raffinose per 100 g of DM, while the high-MWCs averaged 3.03 mg of raffinose per 100 g of DM. Additionally, the low-MWC leaves averaged 0.357 mg of raffinose per 100 g of DM (Table 3).

### 2.4. Principal Component Analysis (PCA)

**Figure 5:**
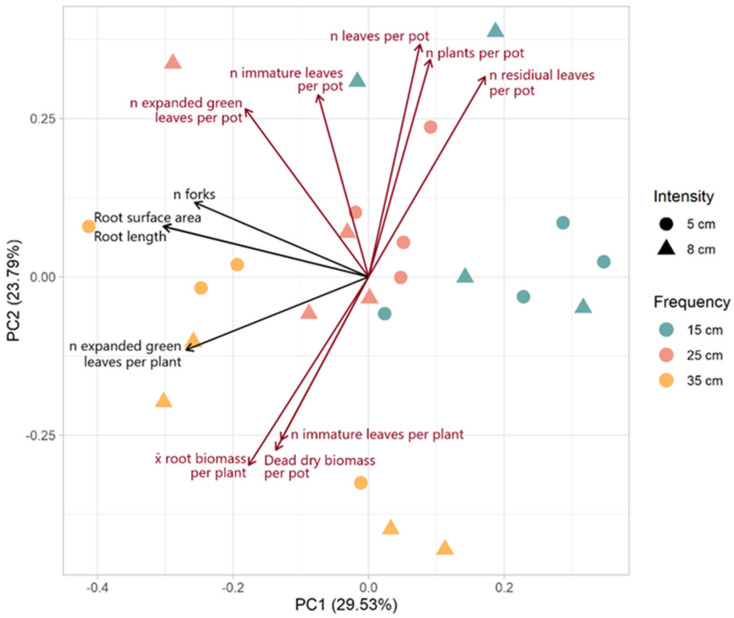
Biplot of PCA of plant characteristics. The principal components (PCs) were calculated with all measured variables of morphological traits, root architecture, and high and low molecular weight carbohydrates. However, loadings are only presented for those variables with loadings greater than 0.25 or less than −0.25 for PC1 (black) or PC2 (dark red).

The biplot of the PCA shows the variables with loadings greater than 0.25 or less than −0.25 for PC1 or PC2 (Figure 5). PC1 explained 29.53% of the variance and was most strongly associated with root architecture (n forks, surface area, and length). PC2 explained 23.79% of the variance and was most strongly associated with above-ground productivity. PC1 could primarily be described as root architecture. The vectors for root surface area, root length, and the number of forks were closely aligned, indicating a strong positive correlation among these variables. The number of expanded green leaves per plant also shared a similar orientation on PC1, suggesting that PC1 may more broadly represent vigor. Variables such as the number of leaves, immature leaves, plants, and the number of residual leaves per pot loaded positively on PC2 are associated with one another and collectively reflect overall leaf production and plant density. The vectors for dead DM per pot and the number of immature leaves per plant loaded negatively on PC2, suggesting that these variables are inversely related to above-ground productivity.

There was a distinct grouping of defoliation frequencies across the PCA biplot, and, indeed, defoliation frequency was significantly associated with both PC1 (F = 9.856, *p* = 0.001) and PC2 (F = 8.440, *p* = 0.003). More frequent defoliation was associated with higher values on PC1 and PC2. Samples subjected to a 15 cm ELL defoliation frequency were clustered neutrally positively along PC1 and positively along PC2. There was a complete separation between this group and the lowest defoliation frequency (35 cm of ELL) group. The middle-frequency group (25 cm of ELL) was clustered between the high (15 cm of ELL) and low (35 cm of ELL) groups around the origin. There was no significant association of intensity with either PC1 (F = 0.213, *p* = 0.648) or PC2 (F = 0.169, *p* = 0.686) and no association of the interaction of frequency and intensity (PC1: F = 1.390, *p* = 0.275; PC2: F = 2.224, *p* = 0.137).

## 3. Discussion

Our results demonstrated that higher defoliation frequencies influenced morphological traits, diminishing the carbohydrate content in leaves and DM mass in both above- and below-ground more than residual height. Therefore, it indicates that under higher defoliation frequencies, the plantain plants reduce their capacity for photosynthesis (less above-ground growth) and for soil exploration, negatively affecting the resource uptake from the soil. These findings suggest that the defoliation frequency can be one of the main reasons for the low persistency and herbage mass production of plantain after the first years of growth in rotational grazing systems.

### 3.1. Morphological Components and Above- and Below-Ground Biomass

The multivariate analysis suggests that at more frequent defoliation (15 cm of ELL), plants utilize resources to maximize above-ground productivity, producing more leaves and plants per pot. At a lower defoliation frequency (35 cm of ELL), plants appeared to assign more resources toward developing root biomass. This aligns with previous findings for plantain pastures [4,21]. Plantain exhibits high phenotypic plasticity in its morpho-physiological and chemical traits [30], allowing it to thrive in diverse environments [31,32] and under severe defoliation regimes [4,23]. The allocation of photoassimilates showed a similar priority to that of grasses, namely: (i) restoration of the photosynthetic area, (ii) replenishment of energy reserves, (iii) tiller initiation, and (iv) root growth. High frequency of defoliation in perennial ryegrass [14], cocksfoot grass [15,16,18], pasture brome [1,17,18], and chicory [11] negatively affect root mass [17], root distribution within the soil profile, and root length [1]. This suggests that consistent with results from all previously named forage species, root growth is likely not a priority for plantain and is only promoted when the photosynthetic restriction is minimal (i.e., under lower defoliation frequencies and intensities) in more advanced leaf development stages. 

These findings indicate that after a threshold for photoassimilate production, the plant starts to allocate more resources to the root system, which is evidenced by the increase in root surface, length, and mean root mass after the defoliation frequency of 25 cm ELL and by the above mass: below mass ratio and root tips at the 8 cm defoliation height. While the effect of defoliation frequency did not significantly affect the number of plants per pot at a 5 cm cutting height, less intensive defoliation resulted in a decrease in the number of plants per pot, likely due to shading at the base of the pots produced by this treatment [14]. In contrast, Lee et al. [4] reported no effect of defoliation frequency nor residual height on plantain swards density (cv. ‘Ceres Tonic’) defoliated at 15, 25, 35, or 45 cm of ELL and at 3–5 or 6–8 cm of residual height over 18 months under field conditions. 

Less frequent defoliation (35 cm of ELL) increased herbage DM mass, suggesting a shift in resource allocation towards more lignified tissues such as dead, dry mass, and stems. The increase in cell wall constituents (lignin, hemicelluloses, and cellulose) under less frequent defoliation can lead to a lower herbage quality for ruminant nutrition, reducing herbage intake and animal production and decreasing herbage use efficiency. In contrast, defoliation at 15 and 25 cm of ELL resulted in a higher leaf count per pot compared to 35 cm of ELL. However, these leaves were predominantly residual, with lower photosynthetic capacity than fully extended leaves. This is consistent with the slower leaf elongation and appearance rates in plants managed at 15 cm ELL [23], resulting in decreased herbage DM per plant compared to less frequently defoliated plants. The increased rate of residual leaf growth in plants cut at 15 cm compared to 25 and 35 cm of ELL, along with the absence of differences in the actual quantum efficiency of PSII between defoliation treatments reported by Merino et al. [23], confirms the adaptability of plantain to severe defoliation management, provided there are no additional stressors. 

Repeated defoliation events affect individual plants by removing photosynthetically active biomass, including leaves and stems. Thus, plants remained mainly vegetative, probably because they were unable to recover a sufficient photosynthetic surface area for reproductive stem investments, as suggested by Lee et al. [21]. The reduction in reproductive stems and dead dry mass enhances the herbage’s nutritional value, characterized by a higher crude protein and reduced neutral detergent fiber concentration [4,23], which should be considered to improve animal performance. Plants cut at 15 cm ELL increased the vegetative generation of new plants, likely related to the activation of dormant meristems and/or the reallocation of assimilates from storage organs to meristems [33]. This demonstrates the ability of plantains to regrow after grazing or mechanical defoliation [34]. However, the failure to generate reproductive stems under field conditions could negatively impact the development of new seedlings, decreasing the long-term persistence of plantain pastures [35].

More severe defoliation could lead to alterations in resource distribution to both above- and below-ground biomass over successive rotations [12,33]. This was evident in the present study, where a decrease in defoliation frequency and residual height corresponded with a decline in root dry mass per plant, as previously reported under greenhouse [11] and field conditions [36]. However, defoliation frequency did not affect root dry mass per pot or the ratio of herbage to root growth per plant. These results suggest that defoliation frequency had a similar influence on below-ground as on above-ground growth, indicating how plantain plants maintain a balanced allocation between above- and below-ground biomass at both the individual and population levels. Thus, this balance is guaranteed by the increase in the number of plants per pot under more frequent defoliation. Regarding the effect of defoliation intensity on the spatial distribution of dry mass, root dry mass per plant increased under the lowest defoliation intensity (defoliation at 8 cm height) and lowest defoliation frequency (defoliation at 35 ELL). 

### 3.2. Root Morphology and Architectural Traits

The reduction in root traits, shoot growth, and energy reserves due to higher defoliation frequency or intensity are linked to the plant’s capacity to produce photoassimilates and its changes in resource allocation according to its developmental stage [1,17], and this response appears to be species dependent. Fine root length was the most sensitive variable related to defoliation frequency. Root growth is one of the latest priorities for photoassimilate allocation after defoliation in grasses [14,17] and plantains [11]. Under frequent defoliation rates, plants prioritize leaf regrowth and WSC storage over root growth [1,14,17]. Therefore, any restriction caused by varying defoliation frequencies and/or intensities will negatively affect root growth to different extents [1,17]. 

The reduced total root length and surface area in plants defoliated at 15 cm of ELL suggests that plantain plants compensate for the continuous loss of above-ground biomass and the amount of carbon captured through photosynthesis at the expense of root growth. Defoliation at 15 and 35 cm and intensity at 5 and 8 cm induced an increase in the average root diameter, indicating a negative effect on water and nutrient uptake by the plant roots. The highest defoliation frequency in plantain plants also negatively affects soil exploration capacity by reducing the length and surface area of fine and coarse roots. Root surface length is the primary determinant of the ability of roots to capture resources from the soil [24], leading to decreased water uptake, nutrient acquisition, and overall resilience to environmental fluctuations [37]. Thus, the effects of defoliation at 15 cm ELL may not be sustainable over the long term if root biomass is insufficient to meet the demands for nutrients and water. 

Under a moderate defoliation frequency (25 cm ELL), plantain develops a more efficient root system for resource capture, showing coordination with the greater production of fully expanded green leaves. Roots with smaller diameters are more efficient at capturing soil resources than thicker roots [24]. This results in greater photosynthetic capacity and DM production compared to plants cut at 35 cm ELL, probably related to a higher proportion of mature leaves, which have higher photosynthetic rates and capacities than immature leaves [38]. 

### 3.3. Impact of Defoliation on Carbohydrate Reserves and Regrowth 

After defoliation, grass species often prioritize recovering leaf area at the expense of WSC reserves to restore photosynthetic capacity following the removal of photosynthetic tissue [14]. Intensive or continuous defoliation leads to insufficient restoration of WSC reserves, resulting in reduced herbage production and pasture persistence [39,40,41]. Prior studies have indicated that plantain root WSC reserves are sensitive to defoliation frequency but unaffected by defoliation intensity. Lee et al. [4] observed a decrease in total WSC reserves 14 days after plantain defoliation, with replenishment to the pre-defoliation level occurring 35 days post-defoliation. Consistent with these findings, our results showed higher levels of high-MWC in leaves cut at 35 cm of ELL approximately 28 days post-defoliation, regardless of residual height. Defoliation at 35 cm ELL resulted in the most fully expanded and immature growing leaves, allowing for a greater photosynthetically active leaf area and replacement of WSC reserves. However, even with elevated WSC levels, root WSC reserves remained unchanged compared to those of plants cut at 15 cm and 25 cm of ELL, consistent with the observations of incomplete reserve replenishment at 14 days post-defoliation described by Lee et al. [4]. 

Low-MWC in leaves and roots showed no differences across defoliation frequencies. Under normal conditions, sucrose and glucose, i.e., direct products of photosynthesis, are stored or transported to sink organs like roots for energy storage [37]. However, defoliation increases the energy demand for regrowth, which in turn limits the transport of nutrients to the roots. Low carbon availability in the 15 cm and 25 cm of ELL defoliation frequency treatments resulted in fewer fully expanded and immature growing leaves, more residual leaves, and lower roots and aerial dry mass per plant. This insufficient supply of high-MWC adversely affected plantain regrowth, corroborated by the negative impacts on photosynthetic pigment content and fluorescence values (F_0_, F_s_, F_m_) reported earlier [23]. Recent research [42] observed a negative correlation between defoliation frequency, starch, and WSC content in plantain roots but found no link with defoliation intensity, which is consistent with our findings. Interestingly, they noted that plant regrowth capacity was unrelated to available WSC, which is in contrast to our results. The differences in these findings may arise due to variations in methods used to measure total WSC, where prior research [42] concentrated on starch quantification, while our protocol [4] measured fructans and raffinose family oligosaccharides. Janeček et al. [43] identified that the main high-MWC in plantain roots are raffinose family members, while starch is present in low concentrations. Raffinose family oligosaccharides are considered both storage carbohydrates and stress protectants [44]. Thus, their presence in both above- and below-ground organs of *P. lanceolata* under defoliation stress is expected. 

## 4. Materials and Methods

### 4.1. Site Description

The research project was conducted for nine months in a plant growth chamber measuring 19 m^2^ at the Laboratory of Forage Species Management at the Universidad de Concepción in Chile. The temperature within the chamber was maintained at 20 ± 2 °C daily, while the relative humidity was kept within a range of 45% to 60%. These conditions were monitored using a thermochron data logger (iButtons DS1923, Maxim Integrated Products Inc., San Jose, CA, USA). Lighting conditions were carefully managed to establish a 14 h light and 10 h dark cycle, employing two 1000-watt LED systems (30 cm × 50 cm, ECO LED-1000, ProGarden, Dongguan, China) with a photosynthetic photon flux of 150–170 µmol m^−2^ s^−1^. The light spectrum was composed of the following proportions: 68% at 660 nm, 12% at 460 nm, 14% at white light, 4% at 730 nm, and 2% at 410 nm. To achieve a photosynthetic active radiation (PAR) of 400 µmol m^−2^ s^−1^ at the plant level (referred to as moderate PAR condition by Neugart et al. [45], the distance between the light panels and the ground level of the pots was adjusted accordingly. Additionally, a quantum sensor (SQ 521, Apogee, Logan, UT, USA) was employed to measure PAR, determining 100 µmol m^−2^ s^−1^ at ground level [46].

### 4.2. Treatments and Experimental Design

The study involved six treatments conducted in a completely randomized experimental design. The treatments were organized in a factorial arrangement, combining three distinct defoliation frequencies (determined by the time it took for leaves to reach lengths of 15, 25, and 35 cm) and two defoliation intensities (5 cm and 8 cm of residual heights). Each treatment was replicated four times, resulting in 24 pots.

### 4.3. Plant Establishment and Management

Plantain plants (cv. ‘Ceres Tonic’) were successfully grown in plastic pots (25 × 25 × 27 cm), serving as the experimental units. The seeds were germinated in a 10 cm diameter Petri dish. A week later, once the radicles reached 0.5 to 2.0 cm in length, the plantain seedlings were transplanted into a 50 alveoli plant nursery with a height of 10 cm. After three weeks, four seedlings were redistributed evenly among each pot, containing 1.5 kg of dry soil composed of 80% peat (Kekkila Professional), 10% perlite, and 10% vermiculite. After one month, all pots were thoroughly irrigated, and their saturated weights were recorded following Earl’s methodology [47] to establish the irrigation protocol. This process was repeated on June 4 to accommodate the increase in pot weight due to root biomass growth.

Defoliation treatments began after three weeks when all plants had grown at least six fully developed leaves [48]. To ensure uniformity among the pots, they were carefully matched based on the number and height of mature leaves per plant. Each plant exhibited 13.5 mature leaves and 22.4 cm of leaves height on average. Subsequently, the pots were randomly assigned to one of six defoliation treatment groups. The plants were irrigated weekly, and a soluble fertilizer plant food (Phostrogen; 13% N, 10% P, 27% K, plus trace elements) was applied at a concentration of 2.8 g L^−1^ to maintain sufficient nutrient levels. Throughout the experiment, moisture content in the pots was maintained between 75% and 100% of their field capacity (water-holding capacity). The pots were weighed weekly using a precision balance (DY208, Maigas, China) with a capacity of 30 kg and an accuracy of 0.5 g. The required amount of water was added weekly to sustain a consistent pot weight corresponding to the target field capacity (approximately 400 mL per pot). These management practices were consistently upheld throughout the entire duration of the experiment.

Every three days, the ELL of one leaf per plant, selected randomly from the most recently fully extended leaves, was measured (16 leaves per treatment, totaling 96 leaves) using a ruler to measure from the base to the apex of the leaf. Once the average of ELL reached the specified height (15, 25, or 35 cm) per treatment, the plant material in each pot was harvested by cutting at 5 or 8 cm residual height using a hand-shear and shear. Plants defoliated at 15 and 25 cm were cut on average every 10 and 15 days, while the average of the 35 cm treatment was 26 days [23].

### 4.4. Morphological and Biochemical Measurements

At the conclusion of the study, a comprehensive assessment was conducted to compare the impacts of different treatments on several aspects: above-ground biomass, allocation of carbohydrate reserves, and root morphology and structure. Various observations and measurements were taken across all pots. The following records were made, and averages calculated: (i) the count of plants, different leaf types (fully expanded green leaves, immature growing leaves, residual leaves), and stems per plant; (ii) measurement of the length of fully extended green leaves per plant; and (iii) recording of the number of plants, leaves, stems, shoots, and seedlings per pot. To ensure consistent evaluation, the herbage was uniformly cut at ground level in the afternoon hours (from 1300 to 1800 h) to avoid diurnal variations in WSC concentration [49]. The harvested herbage was sorted into leaves, stems, and dead material to determine their proportional contribution to the total DM. One leaf sample from each pot (n = 24) was taken at 6 cm height, while the stubble (biomass below 6 cm) was weighed and stored for WSC quantification. The roots (and crown) were harvested, cleaned using a 20% chlorine bleach solution to remove visible root tissue, and manually recovered through a 1 mm sieve. Subsequently, the residual herbage and root samples were dried in a forced air oven (BOV V-125F, BioBase, Jinan, China) at 60 °C until a constant weight was attained. The samples were weighed, ground to 1 mm, and stored in paper bags. 

The root trait assessments were conducted on one plant per pot (n = 24). The dry root samples were arranged evenly in a transparent tray (30 cm × 20 cm) with a 1 cm layer of water and scanned at a resolution of 200 dpi (dots per inch) using a scanner (Epson Expression 12000XL, Nagano, Japan) equipped with a double light source to prevent root overlap. The scanned images were subsequently analyzed for several root variables such as morphological (i.e., total root length, average root diameter, root surface area, and root volume) and architectural traits such as number of tips (i.e., elongation points), forks (i.e., number of root bifurcations), and crossings (i.e., overlapping parts) by using WinRHIZO Reg software (V7.0, Regent Instrument Inc., Quebec, QC, Canada). After scanning, the top 10 cm of each root was dried at 60 °C for WSC analysis. Finally, biomass per plant (above and below ground level) was measured based on DM weight.

The vessel elements from each treatment were measured by dissociating the roots (n = 3 roots for each treatment) using 80% sodium hypochlorite. The segments of 1 cm in length of the main roots from each dehydrated sample were immersed in the hypochlorite solution until they became transparent. The root segments were then thoroughly rinsed in tap water (3 times) and distilled water (3 times) and stained in a 5% ethanolic safranin solution [50]. Subsequently, three aliquots were taken from each sample using a Pasteur pipette, placed on a slide, and then covered with a coverslip. In each sample, 15 vessel elements were photographed using a Leica photomicroscope (Leica DM2500, Wetzlar, Germany). The width and length of the vessel elements (n = 15 for each sample) were determined from the images using the ImageJ Program.

The WSCs were extracted from 25 mg of dried and ground plant material following the protocol of Lee et al. [21] using 1 mL 80% ethanol and subsequently 1 mL distilled water. Carbohydrates were classified into low and high molecular weight. The ethanol fraction contains low molecular weight carbohydrates (low-MWC), including glucose, fructose, sucrose, and fructans, whereas the water fraction contains high molecular weight carbohydrates (high-MWC), such as fructans. Carbohydrate concentration was determined using the phenol-sulfuric acid method [51], utilizing raffinose pentahydrate (≥99% purity, CAS 17629-30-0, Sigma Aldrich, St. Louis, MO, USA) as a calibration standard. Raffinose is the main WSC reserve of plantain [43]. The absorbance was measured using a Synergy H1M Microplate Reader (BioTek SH1MF, Santa Clara, CA, USA), and the results were expressed as grams of raffinose per 100 g of dry matter (g 100 g^−1^ DM). 

### 4.5. Statistical Analysis

#### 4.5.1. Univariate Analysis

Data were subjected to a two-way analysis of variance with pot as the experimental unit and defoliation frequency and intensity as fixed effects. Differences between groups were considered statistically significant at *p* < 0.05. Fisher’s least significant difference (LSD) was used for the statistical separation of means when the ANOVA results were significant (*p* < 0.05). Statistical analysis was performed using GenStat (22nd Edition) software.

If the data were not normally distributed, as was the case for vessel elements, data were transformed with the natural logarithm, or non-parametric statistics were used (box plots, mean, median, percentiles, and standard deviation). The Kruskal-Wallis’s test (*p* < 0.05) was used to compare the means of the treatments (for not normal distribution).

#### 4.5.2. Multivariate Analysis

To elucidate the relationships among the various growth variables and treatments, we performed a Principal Component Analysis (PCA). The PCA was performed using all the measured variables for morphological traits, root architecture, and high and low molecular weight carbohydrates. While the PCA was calculated using all those variables, due to the large number of variables, loadings are only presented for those variables with loadings greater than 0.25 or less than −0.25 for the first principal component (PC1) or second principal component (PC2). The frequency, intensity, and their interaction were tested for significant associations with PC1 and PC2 using an aligned rank transformation analysis of variance (ART ANOVA). PCA, ART ANOVA, and figures were performed in R (i386 4.1.2) and R Studio (2021.09.1) using packages ARTool, ggplot2, ggfortify, and Cairo.

## 5. Conclusions

Defoliation frequency significantly impacts the morphological traits and water-soluble carbohydrate content of *P. lanceolata* plants, affecting dry mass distribution above and below ground levels. The most frequent defoliation (every 15 cm of extended leaf height) negatively impacts the plantain plant’s dry mass by reducing aerial growth, root length, and surface area. In contrast, less frequent defoliation (every 35 cm) favors leaf growth and root biomass development, increasing water-soluble carbohydrate levels in leaves, indicating an adaptive strategy for resource acquisition efficiency and carbohydrate replenishment. Nevertheless, root reserve concentration remains unchanged between defoliation frequency treatments, potentially influencing plant recovery capacity and resilience to environmental fluctuations. 

Defoliation at a residual height of 5 cm resulted in significant reductions in root dry mass and overall plant growth compared to defoliation at 8 cm. More frequent defoliation produced fewer root tips and shorter, smaller roots, negatively affecting the plant’s ability to capture water and nutrients. In contrast, defoliation at 8 cm allowed for better root development and a more balanced allocation of resources, leading to improved plant growth and higher photosynthetic efficiency. Specifically, plants defoliated at a residual height of 5 cm showed a 32% increase in the ratio of above-ground to root biomass compared to those defoliated at 8 cm.

Based on these insights, defoliations with a frequency of 25 cm extended leaf height (approximately every 15 days) and a residual height of 8 cm are recommended for optimizing *P. lanceolata* grazing management. This management promotes a greater proportion of leaves, balancing herbage production and root development, and ensures high nutritional value and sustainable carbohydrate use. Understanding how defoliation frequency and intensity influence morphophysiological and chemical responses in *P. lanceolata* can enhance the sustainability of pastoral production systems. Therefore, this management strategy could improve pasture resilience and support more sustainable livestock production. It is particularly important to understand the response of plantains to defoliation under field conditions, especially in the context of climate change, which exacerbates soil moisture deficits and challenges traditional forage systems. This aspect needs to be thoroughly evaluated in future research.

## Figures and Tables

**Table 1:** Morphological traits of the above-ground biomass of plants of *Plantago lanceolata* submitted to three defoliation frequencies (15, 25, and 35 cm of extended leaf length) and two defoliation intensities (5 and 8 cm).

	Frequency (F)			Intensity (I)			*p* Value		
Variable	15	25	35	5	8	SEM	F	I	F × I
Number of plants pot^−1^ *	6.16 a	4.77 ab	2.56 b	4.46	4.26	0.218	**0.003**	0.794	**0.028**
Total number of leaves pot^−1^	70.6 a	65.9 a	35.0 b	58.5	55.8	11.22	**0.010**	0.774	0.160
Number of stems pot^−1^	3.5 b	10.8 a	13.5 a	9.8	8.8	2.52	**0.003**	0.633	0.100
Number of residual leaves pot^−1^ *	38.9 a	29.9 a	7.9 b	22.0	20.0	1.30	**<0.001**	0.654	0.271
Number of fully expanded green leaves pot^−1^	12.5	18.6	14.1	15.9	14.2	3.01	0.137	0.506	0.172
Number of immature growing leaves pot^−1^ *	12.4	14.1	10.5	13.0	11.4	1.18	0.234	0.339	**0.001**
Leaves dry mass pot^−1^ (g)	1.46 b	2.54 a	1.81 ab	2.08	1.79	0.354	**0.021**	0.318	0.125
Stems dry mass pot^−1^ (g) *	0.023 b	0.178 a	0.300 a	0.125	0.157	0.0102	**0.003**	0.607	0.198
Dead dry mass pot^−1^ (g) *	0.551 c	0.838 b	2.992 a	1.007	1.233	1.1708	**<0.001**	0.132	0.811
Roots dry mass pot^−1^ (g)	0.667	0.937	0.825	0.784	0.836	0.1136	0.084	0.582	0.131
Number of residual leaves plant^−1^ *	6.65 a	6.40 a	3.36 b	5.08	5.38	0.074	**0.001**	0.681	**0.044**
Number of fully expanded green leaves plant^−1^	2.12 b	3.97 a	4.97 a	3.65	3.72	0.596	**<0.001**	0.880	0.165
Number of immature growing leaves plant^−1^ *	2.11 c	3.00 b	4.44 a	3.01	3.08	1.148	**<0.001**	0.834	0.769
Mean above dry mass plant^−1^ (g)	0.256 b	0.554 a	0.632 a	0.482	0.479	0.0910	**0.002**	0.962	0.512
Mean root dry mass plant^−1^ (g)	0.113 c	0.203 b	0.341 a	0.188	0.251 a	0.0242 b	**<0.001**	**0.005**	**0.012**
Above mass: below mass ratio	2.28	2.75	2.04	2.68	2.03	0.322	0.110	**0.025**	0.369

abc: Different letters within a row, under the Frequency factor, indicate differences in mean values (*p*-value in bold). * Variables with back-transformed mean and standard error of the mean (SEM).

**Table 2:** Root architectural traits of *Plantago lanceolata* plants submitted to three defoliation frequencies (15, 25, and 35 cm of extended leaf length) and two defoliation intensities (5 and 8 cm).

Variables	Frequency (F)			Intensity (I)		SEM	*p*-Value		
Overall Values	15	25	35	5	8		F	I	F × I
Total root length (cm)	338 b	537 a	586 a	459	515	112.8	**0.014**	0.407	0.121
Surface area (cm^2^) *	45.8 c	65.8 b	72.3 a	57.9	62.7	1.20	**0.047**	0.600	0.558
Average root diameter (mm)	0.474	0.407	0.421	0.461	0.407	0.038	0.214	0.105	**0.049**
Root volume (cm^3^) *	0.528	0.659	0.759	0.653	0.631	1.262	0.318	0.857	0.996
Number of crossings *	269	327	375	286	359	1.4	0.639	0.438	0.428
Number of forks *	1995	2535	2786	2234	2612	1.3	0.447	0.484	0.456
Number of tips	1166	1234	1395	1104	1426	236.0	0.618	0.112	0.281
*Values by root thinness*									
% Tips 0 to 0.5 mm	99.0	98.9	98.8	98.9	98.9	0.20	0.829	0.811	0.963
% Tips 0.5 to 1.0 mm	0.826	0.899	1.019	0.832	0.997	0.167	0.524	0.244	0.428
% Tips > 1.0 mm	0.223	0.184	0.149	0.248	0.123	0.0953	0.741	0.124	0.226
Number of tips 0 to 0.5 mm	1155	1221	1379	1093	1411	235.0	0.626	0.114	0.286
Number of tips 0.5 to 1.0 mm	8.62	11.5	13.5	9.17 b	13.25 a	1.91	0.062	**0.018**	**0.028**
Length (cm) roots 0 to 0.5 mm	283 b	461 a	491 a	385	438	66.5	**0.012**	0.350	0.061
Length (cm) roots 0.5 to 1.0 mm	40.2 b	65.4 ab	83.9 a	61.7	64.7	15.8	**0.040**	0.818	0.793
Surface area (cm^2^) roots 0 to 0.5 mm	19.6 b	32.8 a	35.1 a	28.3	30.1	4.84	**0.010**	0.643	0.089
Surface area (cm^2^) roots 0.5 to 1.0 mm	8.2 b	13.2 a	16.8 a	12.3	13.2	3.24	**0.050**	0.732	0.826
Volume (cm^3^) roots 0 to 0.5 mm	0.135 b	0.229 a	0.251 a	0.204	0.207	0.0355	**0.010**	0.916	0.130
Volume (cm^3^) roots 0.5 to 1.0 mm	0.138	0.220	0.278	0.202	0.223	0.0550	0.063	0.643	0.860

abc: Different letters within a row, under the Frequency factor, indicate differences in mean values (*p*-value in bold). * Variables with back-transformed mean and standard error of the mean (SEM).

**Table 3:** High and low molecular weight carbohydrates (MWCs) concentration (mg of raffinose 100 g^−1^ DM) of leaves and roots of *Plantago lanceolata* plants under three defoliation frequencies (15, 25, and 35 cm of extended leaf length) and two defoliation intensities (5 and 8 cm).

	Frequency (F)			Intensity (I)		SEM	*p*-Value		
Variable	15	25	35	5	8		F	I	F × I
Leaf high-MWC concentration	2.80 b	2.75 b	3.94 a	3.32	3.01	0.490	**0.044**	0.452	0.816
Root high-MWC concentration *	3.39	2.55	3.22	2.92	3.14	1.198	0.269	0.640	0.400
Leaf low-MWC concentration	1.15	1.26	1.45	1.23	1.34	0.302	0.621	0.675	0.209
Root low-MWC concentration	0.32	0.39	0.36	0.33	0.39	0.066	0.512	0.252	0.618

ab: Different letters within a row, under the Frequency factor, indicate differences in mean values (least significant difference (LSD), *p* ≤ 0.05, *p*-value in bold). * Variables with back-transformed mean and standard error of the mean (SEM).

## Data Availability

The original contributions presented in the study are included in the article/Supplementary Material; further inquiries can be directed to the corresponding authors.

## Supplementary Materials

The following supporting information can be downloaded at: https://www.mdpi.com/article/10.3390/plants13192773/s1, Figure S1: Number of tips per diameter class (mm); Figure S2: Root length (cm) per diameter class (mm); Figure S3: Root surface area (cm^2^) per diameter class (mm); Figure S4: Root volume (cm^3^) per diameter class (mm).
