# Supplementary figures and images for: Distribution of Non-Structural Carbohydrates and Root Structure of Plantago lanceolata L. under Different Defoliation Frequencies and Intensities
Source: Plants (Basel). 2024 Oct 3;13(19):2773. doi: 10.3390/plants13192773 (PMC11478639; doi:10.3390/plants13192773)

**Supplementary Materials**

**Figure S1**

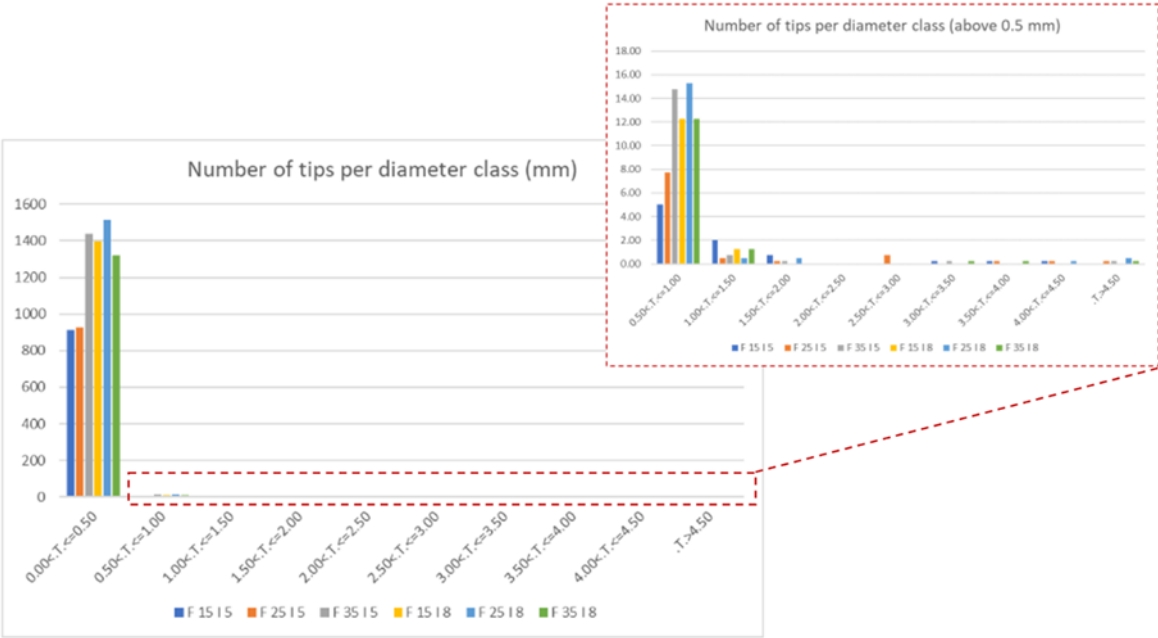

**Figure S2**

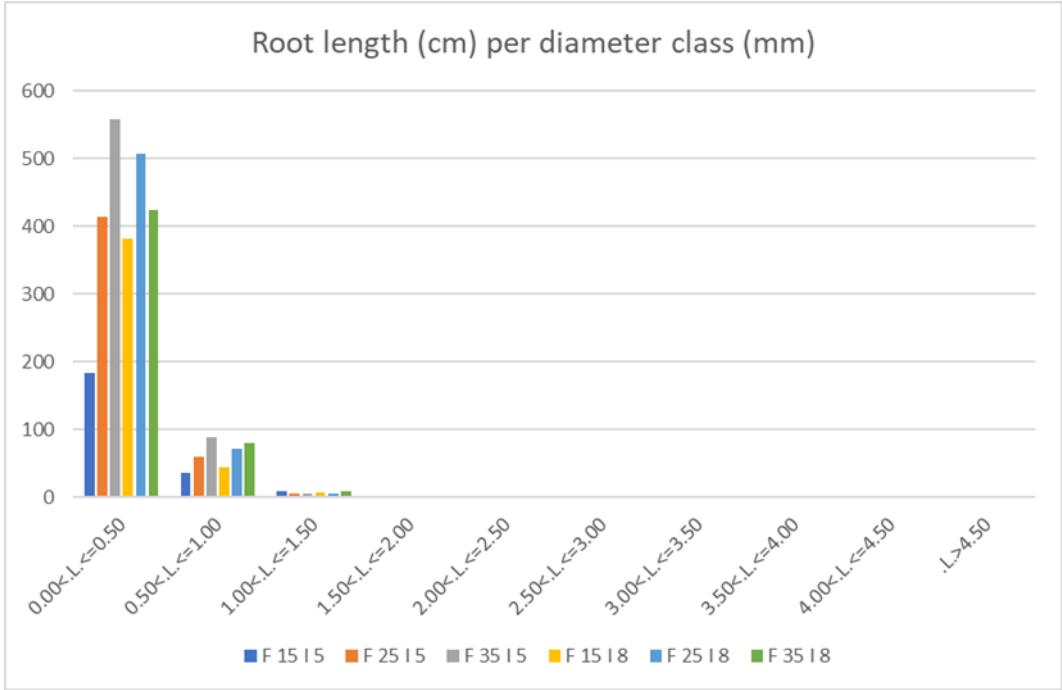

**Figure S3**

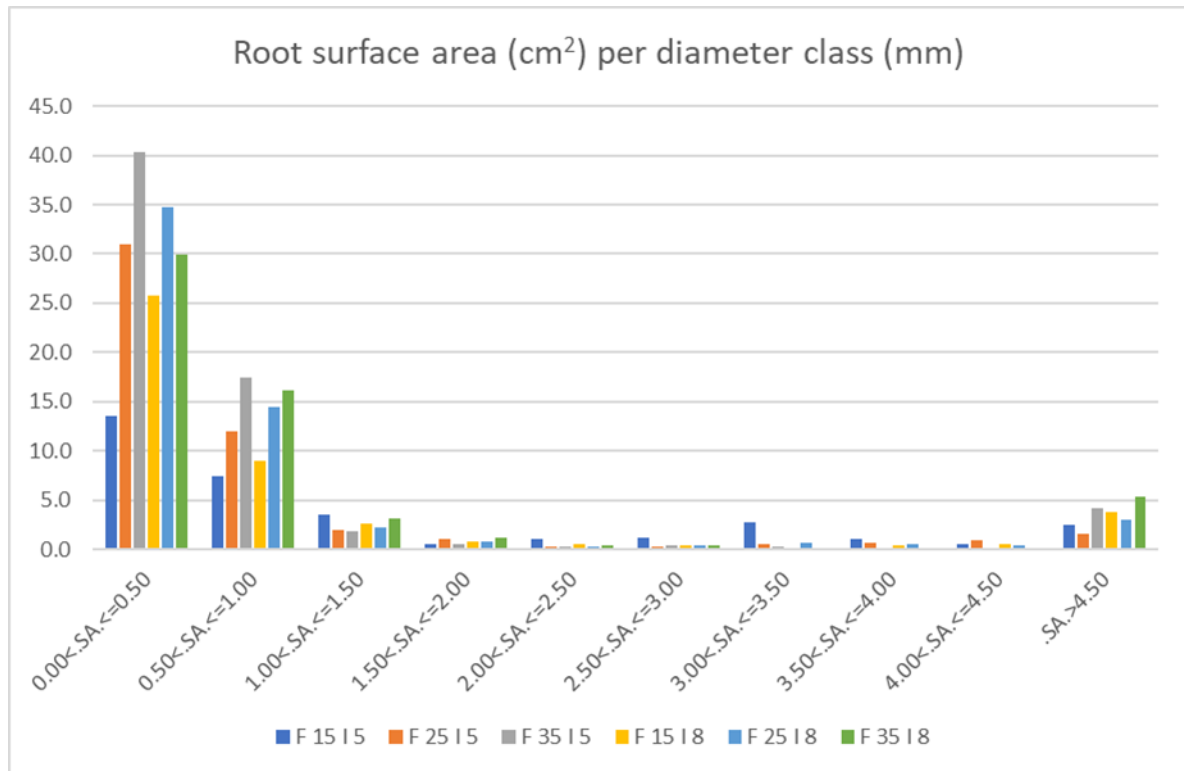

**Figure S4**

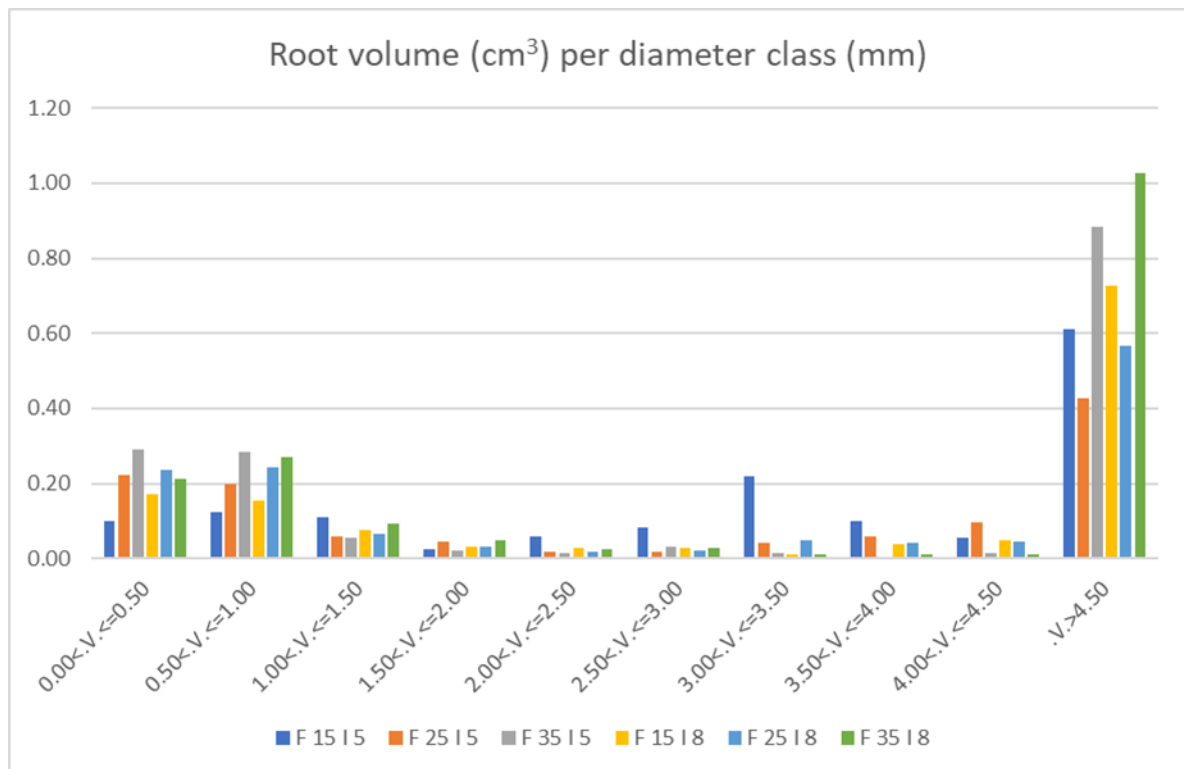

Supplement: Supplementary file 1 [file plants-13-02773-s001.zip › plants-3181994-supplementary.pdf]
